# Incidence of and risk factors for mortality in children with mushroom poisoning

**DOI:** 10.1186/s12245-025-00906-3

**Published:** 2025-06-17

**Authors:** Jie Cheng, Ya Liu, Shaojun Li, Kaibin Pu, Junming Huo, Liping Tan

**Affiliations:** 1 Department of Emergency, National Clinical Research Center for Child Health and Disorders, Ministry of Education Key Laboratory of Child Development and Disorders, Chongqing Key Laboratory of Pediatrics, Children’s Hospital of Chongqing Medical University, No. 136, 2nd Zhongshan Rd, Yuzhong District, Chongqing, 401122 China; 2 Department of Pediatrics, Chongqing Youyoubaobei Women and Children’s Hospital, No. 999, Jiarong Road, Yubei District, Chongqing, 401147 China; 3 Department of Critical Care Medicine, National Clinical Research Center for Child Health and Disorders, Ministry of Education Key Laboratory of Child Development and Disorders, China International Science and Technology Cooperation Base of Child Development and Critical Disorders, Chongqing Key Laboratory of Pediatrics, Children’s Hospital of Chongqing Medical University, Chongqing, 401122 China

**Keywords:** Incidence, Risk factors, Mushroom poisoning, Mortality, Children

## Abstract

**Objective:**

We aimed to evaluate the incidence of and risk factors for mortality in children with mushroom poisoning.

**Methods:**

Sixty-seven children with mushroom poisoning who were hospitalized at the Children’s Hospital of Chongqing Medical University were retrospectively enrolled. The clinical characteristics of the children in the surviving and non-surviving groups were compared. Variables with a *P* value < 0.1 in the univariate logistic regression analysis were included in the multivariate logistic regression analysis. A receiver operating characteristic (ROC) curve was generated to determine the optimal cutoff point.

**Results:**

The mortality rate of children with mushroom poisoning was 23.88% (16/67), and the incidence of death during hospitalization was 35.02 per 1,000 person-days. The median pediatric sequential organ failure assessment (pSOFA) score was 1.00 (interquartile range [IQR] 0.00–3.00). Logistic regression analysis revealed that the pSOFA score was independently associated with mortality (odds ratio [OR] 4.92, 95% confidence interval [CI] 1.59–62.21; *P* = 0.040). The optimal cutoff point of the pSOFA score for predicting mortality was 2.00, with an area under the curve (AUC) of 0.84 (95% CI 0.71–0.88, *P* < 0.001*).

**Conclusions:**

In this study, the incidence of death among children with mushroom poisoning was retrospectively evaluated. The pSOFA score may serve as a good prognostic indicator in children with mushroom poisoning, and children with a pSOFA score ≥ 2 have a significantly increased risk of mortality.

**Supplementary Information:**

The online version contains supplementary material available at 10.1186/s12245-025-00906-3.

## Introduction

Wild mushroom is a widely eaten food worldwide because of its savory flavor [1], and it is estimated that approximately 100 people die from mushroom poisoning every year worldwide [2]. By the end of 2022, more than 500 poisonous mushrooms that have been discovered in China [3]. Mushroom poisoning mostly occurs in Southwest China in the summer and autumn and remains one of China’s most serious food safety problems [3]. Mushroom poisoning may induce damage to multiple organs [2]. From 2010 to 2022, the overall mortality rate of mushroom poisoning ranged from 2.10 to 7.85% [1, 3, 4]. With the increasing education on mushroom poisoning, the focus on identifying mushroom species, and the improvements in treatments, the mortality of mushroom poisoning significantly decreased between 2010 and 2022 [1, 3]. Mushroom poisoning is still an urgent food safety issue in China [3]. The type of mushroom a patient with mushroom poisoning has consumed is not always known [5]; however, knowing the specific type of mushroom a patient consumes is crucial for diagnosis and treatment. However, few studies have reported on mushroom poisoning in children, and the development of an appropriate prognostic indicator is expected to help clinicians evaluate the prognosis. The pediatric sequential organ failure assessment (pSOFA) scoring system [6] consists of the evaluation of five systems (respiratory, coagulation, cardiovascular, neurologic, and renal). These child-specific parameters may help us accurately identify the risk of poisoning-related organ dysfunction and are in line with the pathological characteristics of multi-organ damage in children with mushroom poisoning [7–10].

In this study, we investigated the incidence of death and explored risk factors for mortality in children with mushroom poisoning.

## Methods

### Study designs and patients

This retrospective, observational, cohort study was conducted at the Children’s Hospital of Chongqing Medical University in Chongqing, a National Regional Children’s Medical Center, and National Clinical Research Center for Child Health and Disorders in China (website: https://www.chcmu.com/chcmuy/yygk/yyjj.htm). From January 2012 to December 2023, hospitalized children with mushroom poisoning were enrolled in our study. The inclusion criteria were as follows: (i) hospitalized children; (ii) children with mushroom poisoning. Children with incomplete clinical data were excluded. This study was approved by the Ethics Committee of Children’s Hospital of Chongqing Medical University. File No. 2023 (538). Informed consent was waived by the Ethics Committee of Children’s Hospital of Chongqing Medical University. The relevant guidelines and regulations performed all methods.

### Data collection and definitions

We retrospectively collected demographic data (age, sex), admission patterns, medical records, laboratory tests, therapies, pSOFA scores, and outcomes. The pSFOA score was collected within 48 h of admission. Mushroom poisoning was diagnosed according to the description of the mushroom consumed and the symptoms and clinical findings of the patients [5]. All children who meet the indications for artificial liver treatment will start artificial liver treatment on the day of admission.

### Statistical analysis

Continuous and categorical variables are medians (quartiles) and frequencies (%). Categorical variables were compared with the χ^2^ test or Fisher’s exact test, and continuous variables were compared with Student’s t test or the Mann − Whitney U test. Variables with a *P* value < 0.1 in the univariate logistic regression analysis were included in the multivariate logistic regression analysis. A receiver operating characteristic (ROC) curve was generated to determine the optimal cutoff point of the pSOFA score for predicting mortality in children with mushroom poisoning. An AUC between 0.7 and 0.9 indicated moderate predictive ability [11, 12]. All the statistical analyses were conducted in R software (version 4.3.0). A *P* value ≤ 0.05 was considered to indicate statistical significance.

## Results

### Study population

**Fig. 1:**
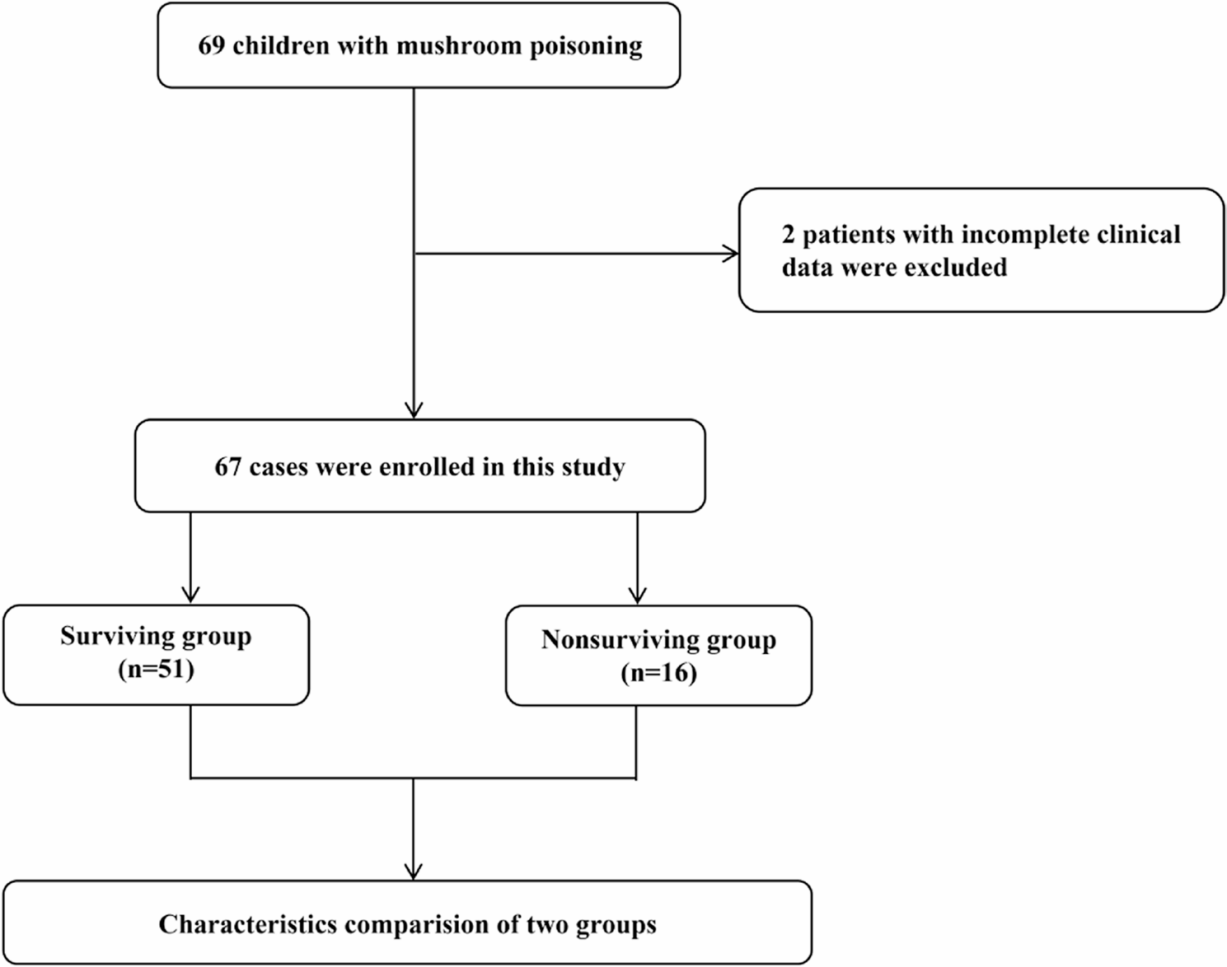
Flow chart of the population

From January 2012 to December 2023, 69 children with mushroom poisoning were admitted to the Children’s Hospital of Chongqing Medical University. Two patients with incomplete clinical data were excluded. Finally, sixty-seven children with mushroom poisoning were enrolled in our study (Fig. 1).

### Clinical characteristics of 67 children with mushroom poisoning

**Fig. 2:**
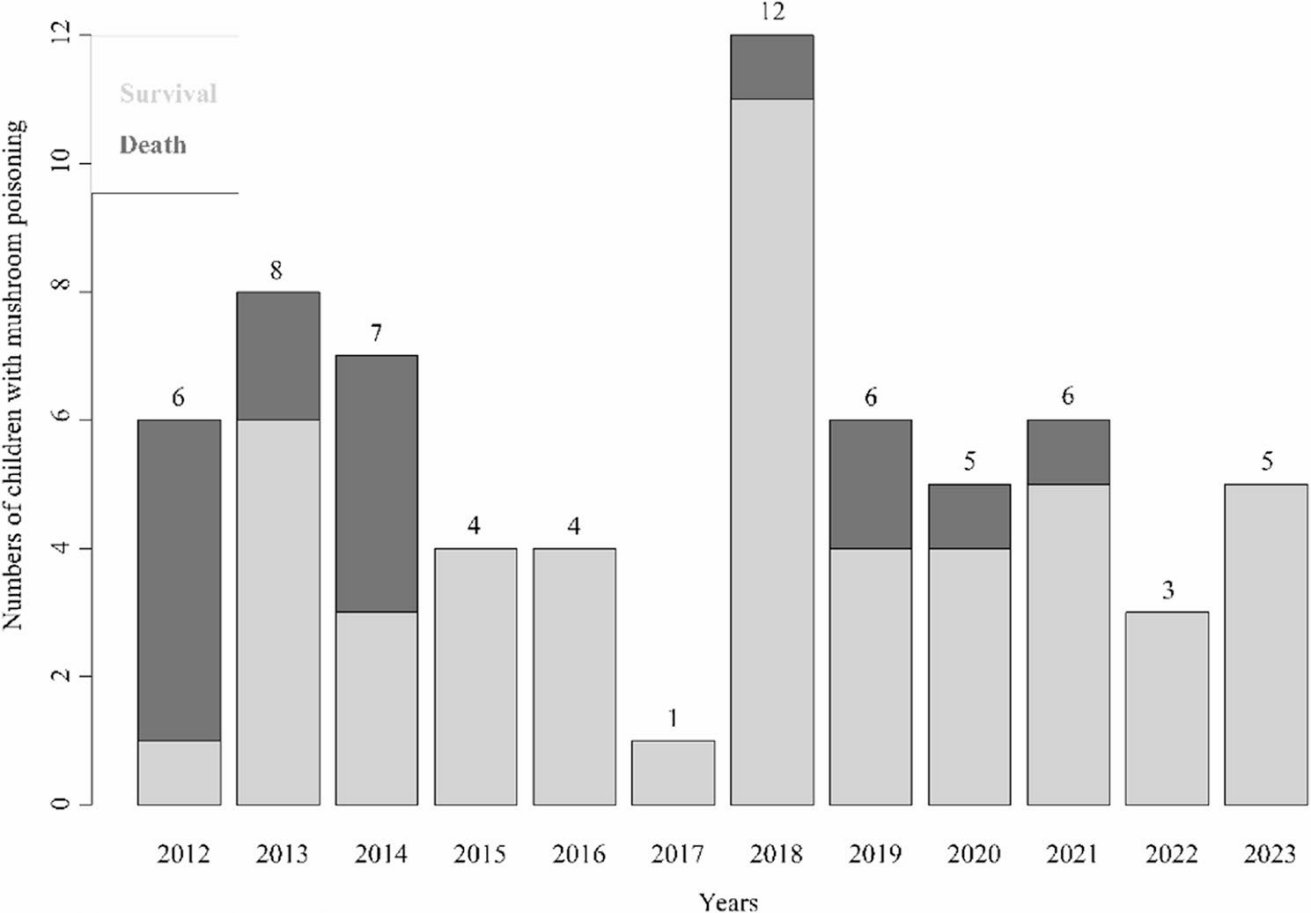
Number of children with mushroom poisoning in different years

The percentage of patients with mushroom poisoning did not significantly change from 2012 to 2023 (Fig. 2). The median age was 7.92 (interquartile range [IQR] 5.67–9.83) years, and 55.22% (37/67) of the patients were male. The median time from consuming mushrooms to admission was 3.00 (IQR 0.33–4.00) days, and the median length of stay was 3.75 (IQR 2.19–10.92) days. Approximately half (30/67, 44.78%) of the patients were admitted from the emergency department, and nearly one-third (22/67, 32.84%) of the patients were transferred to the wards of our hospital from other hospitals. Most patients experienced abdominal pain (35/67, 52.24%), vomiting or diarrhea (63/67, 94.03%), and more than 10% of patients experienced a coma (10/67, 14.93%) or had convulsions (9/67, 13.43%). More than 20% (16/67, 23.88%) of patients needed invasive mechanical ventilation, and approximately two-thirds (43/67, 64.18%) of patients received artificial liver support system therapy. The median pSOFA score was 1.00 (IQR 0.00–3.00). The overall mortality was 23.88% (16/67), and the incidence of death during hospitalization was 35.02 per 1,000 person-days. All the details are shown in Table 1.

**Table 1:** Characteristics of the 67 children with mushroom poisoning

Characteristics	Total (*n* = 67)
Demographic data	
Age (years) (median, IQR)	7.92 (5.67–9.83)
Sex (male) (n, %)	37 (55.22%)
Patterns of admission	
Admitted from the emergency department (n, %)	30 (44.78%)
Transferred to wards from other hospitals (n, %)	22 (32.84%)
Clinical manifestations	
Vomiting or diarrhea	63 (94.03%)
Abdomen pain	35 (52.24%)
Coma (n, %)	10 (14.93%)
Convulsions (n, %)	9 (13.43%)
Laboratory tests	
Coagulation function	
PT (s) (median, IQR)	13.30 (11.70−56.75)
APTT (s) (median, IQR)	29.50 (26.00−56.30)
Fibrinogen (g/L) (median, IQR)	1.75 (1.07–2.30)
Liver function	
TBIL (µmol/L) (median, IQR)	18.00 (6.85–60.55)
Albumin (g/L) (median, IQR)	41.30 (31.65–45.60)
ALT (U/L) (median, IQR)	363.10 (25.85−4332.50)
AST (U/L) (median, IQR)	253.00 (29.80−2070.75)
Kidney function	
BUN (mmol/L) (median, IQR)	5.09 (3.77–6.51)
Creatinine (µmol/L) (median, IQR)	39.00 (31.25–50.10)
Advanced life support	
Requiring for invasive mechanical ventilation (n, %)	16 (23.88%)
With artificial liver support system (n, %)	43 (64.18%)
The time from consuming the mushroom to admission (days)	3.00 (0.33−4.00)
Length of stay (days) (median, IQR)	3.75 (2.19–10.92)
pSOFA score (median, IQR)	1.00 (0.00–3.00)
Mortality (n, %)	16 (23.88%)

### Comparisons of the clinical characteristics between the surviving and non-surviving groups of 67 children with mushroom poisoning

The characteristics of the two groups are shown in Table 2. Patients in the survival group were much older than those in the non-survival group (8.25 years (IQR 6.58–10.92 years) vs. 5.63 years (IQR 2.86–7.27 years), *P* = 0.001). There were no significant differences between the survival and the non-survival groups for the patterns of admission, and there were more deceased patients who had experienced a coma than those who survived (37.50% vs. 7.84%, *P* = 0.009). Compared with the survival patients, the non-survival patients had remarkably higher levels of prothrombin time (PT), activated partial thromboplastin time (APTT), total bilirubin (TBIL), alanine transaminase (ALT), and aspartate aminotransferase (AST), and lower fibrinogen and albumin levels. (*P* < 0.001). The blood urea nitrogen (BUN) and creatinine levels were not different between the two groups. The non-surviving group had more patients who needed invasive mechanical ventilation and artificial liver support systems (68.75% vs. 9.80%, *P* < 0.001; 87.50% vs. 56.86%, *P* = 0.053, respectively). The amount of time between consuming mushroom and admission to the hospital was much longer in the non-surviving group than in the surviving group (4.00 days (IQR 3.00–5.00 days) vs. 2.00 days (IQR 0.31–3.00 days), *P* = 0.002). However, the length of stay differed between the non-surviving group and the surviving group (2.50 days (IQR 1.24–4.50 days) vs. 5.42 days (IQR 2.32–13.19 days), respectively, *P* = 0.044). The patients who died had notably higher pSOFA scores than those who survived (3.50 (IQR 2.00–6.00) vs. 0.00 (IQR 0.00–2.00), *P* < 0.001).

**Table 2:** Comparisons of the clinical characteristics between the surviving and non-surviving groups of 67 children with mushroom poisoning

Characteristics	Survival (*n* = 51)	Non-survival (*n* = 16)	*P*-value
Demographic data			
Age (years) (median, IQR)	8.25 (6.58–10.92)	5.63 (2.86–7.27)	0.001*
Sex (male) (n, %)	26 (50.98%)	11 (68.75%)	0.338
Patterns of admission			
Admitted from the emergency department (n, %)	22 (43.14%)	8 (50.00%)	0.847
Transferred to wards from other hospitals (n, %)	16 (31.37%)	6 (37.50%)	0.881
Clinical manifestations			
Vomiting or diarrhea	48 (94.12%)	15 (93.75%)	1.000
Abdomen pain	28 (54.90%)	7 (43.75%)	0.622
Coma (n, %)	4 (7.84%)	6 (37.50%)	0.009*
Convulsions (n, %)	6 (11.76%)	3 (18.75%)	0.437
Laboratory tests			
Coagulation function			
PT (s) (median, IQR)	12.20 (11.45−16.00)	141.70 (55.35–180.00)	< 0.001*
APTT (s) (median, IQR)	28.40 (25.35–30.85)	88.80 (56.50−119.60)	< 0.001*
Fibrinogen (g/L) (median, IQR)	1.95 (1.54–2.43)	0.77 (0.62–1.04)	< 0.001*
Liver function			
TBIL (µmol/L) (median, IQR)	12.90 (6.20–30.40)	79.50 (64.35–95.18)	< 0.001*
Albumin (g/L) (median, IQR)	43.00 (39.10−46.55)	29.30 (25.15–32.90)	< 0.001*
ALT (U/L) (median, IQR)	47.00 (24.55−2071.10)	5408.85 (2781.23−7562.73)	< 0.001*
AST (U/L) (median, IQR)	39.20 (27.90−677.80)	2130.45 (1537.40−7605.50)	< 0.001*
Kidney function			
BUN (mmol/L) (median, IQR)	5.27 (4.09–6.39)	4.55 (3.48–6.69)	0.374
Creatinine (µmol/L) (median, IQR)	39.30 (33.45–50.10)	34.45 (29.38–48.85)	0.293
Advanced life support			
Requiring for invasive mechanical ventilation (n, %)	5 (9.80%)	11 (68.75%)	< 0.001*
With artificial liver support system (n, %)	29 (56.86%)	14 (87.50%)	0.053
The time from consuming the mushroom to admission (days)	2.00 (0.31−3.00)	4.00 (3.00–5.00)	0.002*
Length of stay (days) (median, IQR)	5.42 (2.32–13.19)	2.50 (1.24–4.50)	0.044*
pSOFA score (median, IQR)	0.00 (0.00–2.00)	3.50 (2.00–6.00)	< 0.001*

*With statistical significance, *P* < 0.05

### Risk factors for mortality in 67 children with mushroom poisoning

According to the univariate logistic regression analysis, the serum albumin concentration, age, and fibrinogen level were protective factors against mortality in children with mushroom poisoning (*P* < 0.05). Furthermore, there was a positive correlation between mortality and a longer time from consuming the mushroom to admission, the need for invasive mechanical ventilation, the use of an artificial liver support system, coma, a higher TBIL, a higher PT, a higher APTT, a higher ALT level, a higher AST level and a higher pSOFA score (*P* < 0.05). According to the multivariate regression analysis, only the pSOFA score was independently associated with mortality (odds ratio [OR] 4.92, 95% confidence interval [CI] 1.59–62.21; *P* = 0.040). All the details are shown in Table 3.

**Table 3:** Logistic regression analysis of risk factors for mortality among 67 children with mushroom poisoning

Variables	Univariate analysis			Multivariate analysis		
	OR	95% CI	*P*	OR	95% CI	*P*
pSOFA	1.89	1.39–2.79	< 0.001*	4.92	1.59–62.21	0.040*
Requiring for invasive mechanical ventilation	20.24	5.35–91.68	< 0.001*			
Coma	7.05	1.71–21.32	0.008*			
With artificial liver support system	5.31	1.30–36.09	0.039*			
The time from consuming the mushroom to admission (days)	1.54	1.14–2.18	0.008*			
APTT (s)	1.04	1.02–1.07	< 0.001*			
TBIL (µmol/L)	1.03	1.02–1.05	< 0.001*			
PT (s)	1.02	1.01–1.04	< 0.001*			
ALT (U/L)	1.00003	1.00001–1.00005	< 0.001*			
AST (U/L)	1.00002	1.00001–1.00004	< 0.001*			
Albumin (g/L)	0.81	0.72–0.89	< 0.001*			
Age (years)	0.73	0.58–0.89	0.004*			
Fibrinogen (g/L)	0.05	0.01–0.19	< 0.001*			

*With statistical significance, *P* < 0.05

### The prognostic value of the pSOFA score for predicting mortality in 67 children with mushroom poisoning

The optimal cutoff point of the pSOFA score for the prediction of mortality was 2.00, with an area under the curve (AUC) of 0.84 (95% CI 0.71–0.88, 87.50% sensitivity and 70.59% specificity, *P* < 0.001), which indicates moderate predictive efficacy [13]. The details of the ROC curve are shown in Supplemental Fig. 1. The Kaplan–Meier survival curve showed that patients with a pSOFA score < 2 had a significantly greater survival probability than patients with a pSOFA score ≥ 2 during hospitalization. The details are presented in Supplemental Fig. 2.

## Discussion

Mushroom poisoning is one of China’s most serious food safety problems [3]. However, few studies have reported on mushroom poisoning in children, and identifying a good and convenient prognostic tool for predicting mortality due to mushroom poisoning is urgently needed. In this study, we investigated the gaps in the knowledge regarding mushroom poisoning in children. Mushroom poisoning mostly occurs in Southwest China [3]. Our study was conducted at the Children’s Hospital of Chongqing Medical University, a National Clinical Research Center for Child Health and Disorders, which treats patients throughout Southwest China (website: https://www.chcmu.com/chcmuy/yygk/yyjj.htm), and this may strengthen the universality of the results of our study.

Only 67 children with mushroom poisoning were enrolled in our study from 2012 to July 2023; the mortality rate reached 23.88% (16/67), and the incidence of death during hospitalization was 35.02 per 1,000 person-days. In recent decades, the reported overall mortality of mushroom poisoning in China has ranged from 2.10 to 7.85% [1, 3, 4], much lower than our study’s mortality. This might indicate that children with mushroom poisoning have more serious adverse outcomes than adult patients. Furthermore, as a National Regional Children’s Medical Center and National Clinical Medicine Research Center (website: https://www.chcmu.com/chcmuy/yygk/yyjj.htm), our center treats critically ill children in the region. More than 30% (22/67, 32.85%) of the cases included in this study were difficult/critically ill children transferred from other hospitals. This explains, to a certain extent, the difference in mortality rates between this study and previous studies of mushroom poisoning patients. On the other hand, the inclusion of such cases not only reflects the core role of our center in complex case management, but also ensures that the research cohort is widely representative in terms of disease severity, providing important support for the universality of the conclusions.

Our study revealed that the pSOFA score was positively associated with mortality in children with mushroom poisoning. The possible explanations are as follows. The pSOFA scoring system is used to evaluate the severity of organ dysfunction in sepsis patients [6] and may also serve as a convenient prognostic tool [14]. Wild mushrooms may be toxic because they induce cellular death in the human body’s cells [15], and the intestines, liver, and kidneys are affected the most [16]. Liver function deterioration always occurs after the gastrointestinal symptoms [17]. Apoptotic hepatocytes cannot remove toxic metabolites, and these apoptotic hepatocytes also cannot synthesize coagulation factors, complement, and lipoproteins [18]. As a result, multiple organ dysfunction syndrome may occur, which can be diagnosed using the pSOFA score. In this study, we also found that children with mushroom poisoning who had a pSOFA score ≥ 2 had a significantly increased risk of mortality.

In this study, most children with mushroom poisoning had gastrointestinal symptoms (abdomen pain, vomiting, or diarrhea), which was in accordance with the findings of Sabeel et al. [17]. Compared with surviving patients, patients who died had lower levels of albumin and fibrinogen, and higher levels of TBIL, ALT, AST, PT, and APTT. A possible explanation is as follows. Liver failure always occurs in patients with critical mushroom poisoning and may present as abnormalities in liver function or coagulation function. Hepatocytes that fail to perform their function may induce multiple organ dysfunction syndrome [18], which indicates poor outcomes. Furthermore, patients in the non-surviving group had a longer time from consuming mushrooms to admission than did surviving patients, which may suggest that timely treatment improves the health of children with mushroom poisoning. Mushroom poisoning can lead to acute liver failure [8], and artificial liver support therapy is critical for children with mushroom poisoning [18]. Yang et al. [19] stated that after continuous artificial liver treatment, liver function-related indicators (e.g., ALT and TBIL) of children with mushroom poisoning have significantly improved, and receiving it may reduce the risk of death. Regarding the timing of treatment, all children in our study who met the indications for artificial liver treatment received artificial liver treatment on the day of admission. The “Chinese Clinical Guideline for the Diagnosis and Treatment of Mushroom Poisoning” [20] recommends that patients with fatal mushroom poisoning should undergo blood purification treatment as soon as possible. Therefore, once a child has mushroom poisoning, artificial liver support treatment should be carried out as soon as possible to grasp the timing of treatment, which is extremely important for improving the prognosis of children and reducing mortality.

There were several limitations in our study. First, this was a single-center, retrospective study with a small sample size, and a multi-center, prospective study with a large sample size was expected. Second, another limitation of our study was that different wild mushroom species may induce different adverse outcomes [3], and our study did not discuss the difficulties in reminiscing or identifying the specific types of eaten mushrooms. However, this study may promote education regarding the safety of consuming mushrooms and improve the screening techniques for poisonous substances. Third, given the significant differences in clinical symptoms and severity among different mushroom poisoning patients, we believe that the toxicity levels vary significantly among different patients. However, in clinical treatment, clinicians focus on mushroom type, intake, symptom changes, and dynamic organ damage indicators and do not perform toxicity level testing. Finally, we did not discuss the influence of specific types of artificial liver support system therapy on prognosis. The advantages and disadvantages of different artificial liver support systems remain unknown, and there is no uniform artificial liver support system therapy available for children with mushroom poisoning [18]. Additional studies on artificial liver support systems for mushroom poisoning are expected.

## Conclusions

In this study, the incidence of death among children with mushroom poisoning was retrospectively evaluated. The pSOFA score may serve as a good prognostic indicator in children with mushroom poisoning, and children with a pSOFA score ≥ 2 had a significantly increased risk of mortality.

## Supplementary Information

Supplementary Material 1: Supplemental Figure 1. ROC curve of the ability of the pSOFA score to predict mortality in 67 children with mushroom poisoning. Supplemental Figure 2. Comparison of the survival probability of patients with different pSOFA scores.

## Data Availability

No datasets were generated or analysed during the current study.

## Abbreviations

ALT: Alanine transaminase
APTT: Activated partial thromboplastin time
AST: Aspartate aminotransferase
AUC: Area under the curve
BUN: Blood urea nitrogen
CI: Confidence interval
IQR: Interquartile range
OR: Odds ratio
pSOFA: Pediatric sequential organ failure assessment
PT: Prothrombin time
ROC: Receiver operating characteristic
TBIL: Total bilirubin
