# Supplementary material for: Incidence of and risk factors for mortality in children with mushroom poisoning
Source: Int J Emerg Med. 2025 Jun 17;18:103. doi: 10.1186/s12245-025-00906-3 (PMC12172370; doi:10.1186/s12245-025-00906-3)
Supplement: Supplementary file 1 — Supplementary Material 1: Supplemental Figure 1. ROC curve of the ability of the pSOFA score to predict mortality in 67 children with mushroom poisoning. Supplemental Figure 2. Comparison of the survival probability of patients with different pSOFA scores. [file 12245_2025_906_MOESM1_ESM.docx]

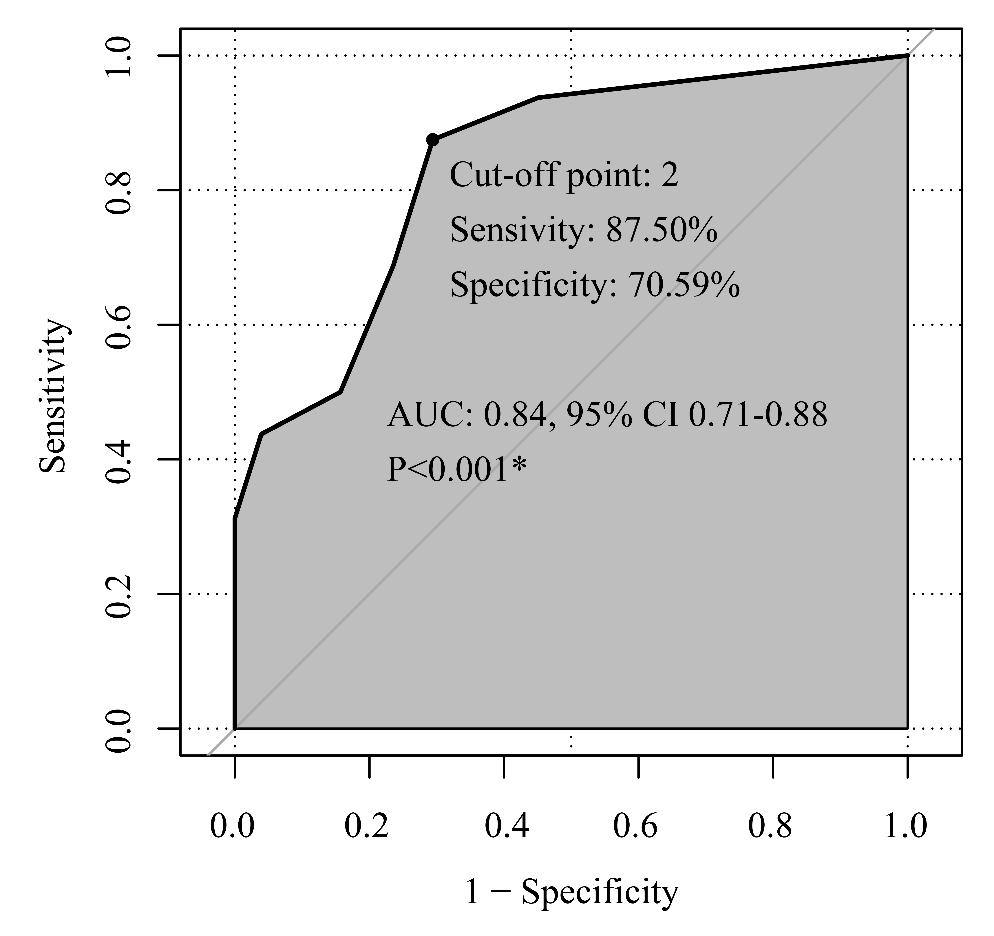


**Supplemental Figure 1 ROC curve of the ability of the pSOFA score to predict mortality in 67 children with mushroom poisoning.**


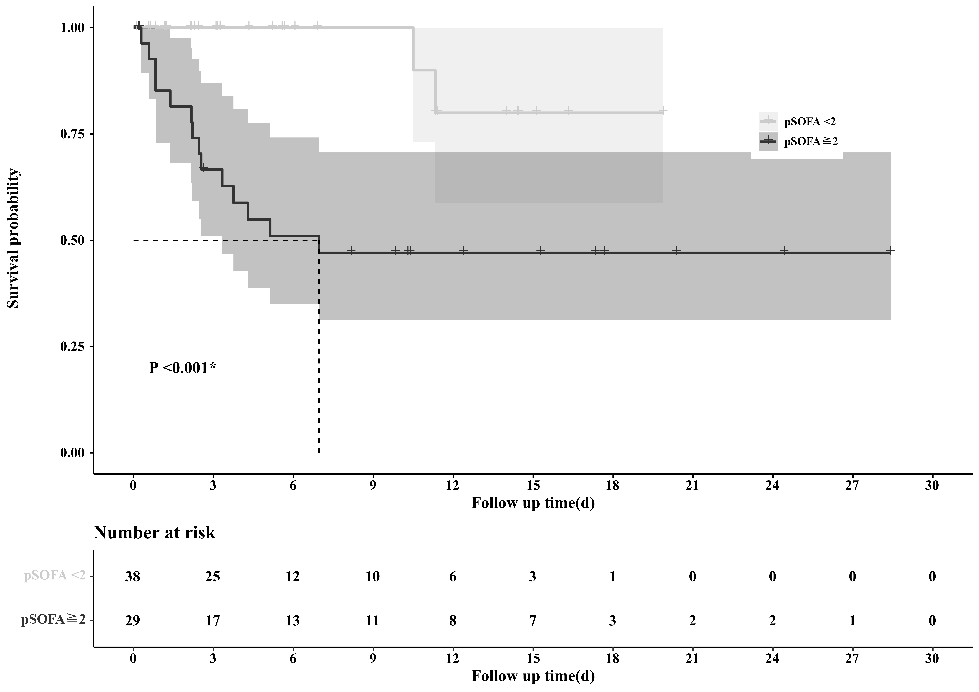


**Supplemental Figure 2 Comparison of the survival probability of patients with different pSOFA scores.**
